# MEIS2C and MEIS2D promote tumor progression via Wnt/β-catenin and hippo/YAP signaling in hepatocellular carcinoma

**DOI:** 10.1186/s13046-019-1417-3

**Published:** 2019-10-17

**Authors:** Lei Guan, Ting Li, Nanping Ai, Wei Wang, Bing He, Yanxia Bai, Zhaocai Yu, Mingyue Li, Shanshan Dong, Qingge Zhu, Xiao Xiao Ding, Shiming Zhang, Ming li, Guangbo Tang, Xiaochun Xia, Jing Zhao, Song lin, Shi yao, Lei zhang, Geng chen, Fang-E Liu, Xinyuan Li, Huqin Zhang

**Affiliations:** 1 The Key Laboratory of Biomedical Information Engineering of Ministry of Education, School of Life Science and Technology, Xi’an Jiaotong University, 28, Xianning West Road, Xi’an, 710049 Shaanxi People’s Republic of China; 2 Department of Ophthalmology, Chinese PLA General Hospital, Beijing, 100853 People’s Republic of China; 3 Department of Immunology, State Key Laboratory of Cancer Biology, Fourth Military Medical University, Xi’an, China; 4 Department of Clinical Pharmacy, University of Michigan, Ann Arbor, MI USA; 5 Department of Otolaryngology-Head-Neck Surgery, The First Affiliated Hospital, Medical School of Xi’an Jiaotong University, Xi’an, 710061 People’s Republic of China; 6 Department of Medical Oncology. Xijing Hospital, The Fourth Military Medical University, Xi’an, 710032 People’s Republic of China; 7 Department of Pathology and Laboratory Medicine, Perelman School of Medicine, University of Pennsylvania, 712 Stellar-Chance Laboratories, 422 Curie Blvd, Philadelphia, PA 19104 USA; 8 School of Electronics and Information Engineering, Xi’an Jiaotong University, Xi’an, 710049 People’s Republic of China; 9 Medical College, Xiamen University, Xiamen, 361102 People’s Republic of China; 10 Department of Medical Technology, Xiamen Medical College, Xiamen, 361023 People’s Republic of China; 11 Department of General Surgery, 967 Hospital of PLA, Dalian, 116041 People’s Republic of China; 12 Medical College, Xi’an Peihua University, Xi’an, People’s Republic of China

**Keywords:** HCC, MEIS2, Alternative splicing, Wnt, Hippo, CDC-73

## Abstract

**Background:**

MEIS2 has been identified as one of the key transcription factors in the gene regulatory network in the development and pathogenesis of human cancers. Our study aims to identify the regulatory mechanisms of MEIS2 in hepatocellular carcinoma (HCC), which could be targeted to develop new therapeutic strategies.

**Methods:**

The variation of MEIS2 levels were assayed in a cohort of HCC patients. The proliferation, clone-formation, migration, and invasion abilities of HCC cells were measured to analyze the effects of MEIS2C and MEIS2D (MEIS2C/D) knockdown with small hairpin RNAs in vitro and in vivo. Chromatin immunoprecipitation (ChIP) was performed to identify MEIS2 binding site. Immunoprecipitation and immunofluorescence assays were employed to detect proteins regulated by MEIS2.

**Results:**

The expression of MEIS2C/D was increased in the HCC specimens when compared with the adjacent noncancerous liver (ANL) tissues. Moreover, MEIS2C/D expression negatively correlated with the prognosis of HCC patients. On the other hand, knockdown of MEIS2C/D could inhibit proliferation and diminish migration and invasion of hepatoma cells in vitro and in vivo. Mechanistically, MESI2C activated Wnt/β-catenin pathway in cooperation with Parafibromin (CDC73), while MEIS2D suppressed Hippo pathway by promoting YAP nuclear translocation via miR-1307-3p/LATS1 axis. Notably, CDC73 could directly either interact with MEIS2C/β-catenin or MEIS2D/YAP complex, depending on its tyrosine-phosphorylation status.

**Conclusions:**

Our studies indicate that MEISC/D promote HCC development via Wnt/β-catenin and Hippo/YAP signaling pathways, highlighting the complex molecular network of MEIS2C/D in HCC pathogenesis. These results suggest that MEISC/D may serve as a potential novel therapeutic target for HCC.

## Background

Hepatocellular carcinoma (HCC) is a primary liver cancer with high mortality. It is one of the most common malignancies in the world, especially in Asia, Africa, and Southern Europe [1]. Although some progress has been made in the study of the pathogenesis of HCC in recent years, the long-term survival rate of HCC patients is still not optimistic. Therefore, further study of the molecular mechanism of liver cancer is needed.

Meis homeobox 2 (MEIS2) belongs to the three amino acid loop extension (TALE) superfamily [2]. TALE homeobox genes represent a subset of the homeobox gene family. TALE genes are crucial for normal development of the nervous system [3–5]. In vertebrates, TALE homeoproteins are represented by the PBX and MEIS/PREP subfamilies [6–8]. Most of them have also been implicated in tumorigenesis. Accumulating research suggests an oncogenic role for MEIS2 in the development of neuroblastoma, leukemia, bladder, prostate, and ovarian cancer [9–14]. It has been shown that MEIS2 is essential for the survival and proliferation of neuroblastoma cell by transcriptional control of M-phase progression [9]. Alternative splicing is important for posttranscriptional regulation of MEIS2. Because of the sequence difference of C-terminal due to alternative splicing, there are two mainly alternatively spliced isoforms of MEIS2, MEISI2A and MEIS2B (MEIS2A/B) and MEISI2C and MEIS2D (MEIS2C/D). It has been reported that MEIS2 affects neuroblastoma proliferation and differentiation in opposing ways, depending on the relative expression of two of its alternative splice isoforms [15]. Nevertheless, the role of MEIS2 in HCC remains poorly defined.

Previous studies have shown that miR-1307 may be a therapeutic target for ovarian cancer [16]. Also, miR-1307 could promote the proliferation of prostate cancer through targeting FOXO3 [17]. MiR-1307-3p is originated from the 3′ end of pre-miR-1307, and increased in breast cancer patients’ serum [18, 19]. However, the upstream regulators of miR-1307 during tumorigenesis and its specific role in HCC remain unclear.

The Hippo signaling pathway inhibition promotes cirrhosis and tumorigenesis in liver [20]. Yes-associated protein (YAP) is a nuclear effector of the cell-density sensing Hippo pathway and it interacts with Src homology phosphotyrosine phosphatase 2 (SHP2), which controls cell proliferation and survival. It has been shown that the tumorigenic role of YAP in HCC requires SHP2. Moreover, on tyrosine dephosphorylation by SHP2, Parafibromin (cell division cycle 73, CDC73) acquires the ability to stably bind to β-catenin. The CDC73/β-catenin complex induces expression of Wnt target genes, which promote HCC progression [21–24]. Nevertheless, the specific regulatory network that controls YAP and SHP2 in HCC still need to be identified.

To investigate the role of MEIS2 in HCC, we examined the expression of MEIS2 in HCC samples and adjacent noncancerous livers (ANL) and found that the level of two alternatively spliced MEIS2 isoform, MEIS2C/D, are up-regulated in HCC tissues. In addition, MEIS2C/D are associated with poor prognosis in HCC patients. Importantly, knockdown of MEIS2C/D could inhibit proliferation and diminish migration and invasion of hepatoma cells in vitro and in vivo. Mechanistically, MEIS2D suppressed Hippo pathway by promoting YAP nuclear translocation via miR-1307-3p/LATS1 axis, while MESI2C activated Wnt/β-catenin pathway in cooperation with CDC73. Interestingly, the interaction between CDC73 and MEIS2C/β-catenin or MEIS2D/YAP depend on its tyrosine-phosphorylation status. Collectively, our studies indicate that MEIS2C/D promote HCC progression and may serve as a novel prognostic biomarker for HCC.

## Methods

### Patients and samples

We collected 118 paired tumorous and adjacent noncancerous liver samples from April 2016 to MAY 2018 in The First Affiliated Hospital of Xi’an Jiaotong University and Xijing Hospital. Detailed clinical pathological parameters were listed in Additional file 1: Table S1. The average age was 52.6 years, and ages range from 38 to 75. A total of 59 males and 59 females were included. There were no patients who received chemotherapy or radiotherapy before surgery excision. Informed consent was obtained from each patient, and the study protocol, which conformed to the ethical guidelines of the 1975 Declaration of Helsinki, was approved by the Institute Research Ethics Committee at The First Affiliated Hospital of Xi’an Jiaotong University and Xijing Hospital.

### Cell culture

HCC-LM3 and MHCC97H cell were routinely culture in DMEM medium supplemented with 1X MEM, 1X Sodium pyruvate, 1X Glutamine + 25 mM HEPES +1X 2-ME (from 55 mM stock 1000X) + 10% FBS and antibiotics. All cultures were maintained in a humidified 5% CO_2_ incubator at 37 °C, and routinely passaged when 80–90% confluent.

### RNA isolation, reverse transcription PCR (RT-PCR) and quantitative real-time RT-PCR (qRT-PCR)

Total RNA was extracted using TRIzol (Invitrogen) according to the manufacturer’s instructions. Reverse transcription was carried out using oligo-dT primers. MiR-1307-3p mRNA levels were detected by using qRT-PCR. 18 s RNA and U6 were used as internal controls. Real-time PCR was performed in an Applied Biosystems 7500 system using Power SYBR Green PCR Master Mix (Applied Biosystems).

### Western blot

The cells were lysed in RIPA buffer in presence of protease inhibitor cocktail (Roche). Whole cell lysates were prepared and subjected to 12% SDS-PAGE and transferred to NC membrane (Bio-Red). The membranes were incubated overnight at 4 °C with the specific antibodies. Membranes were washed and incubated for 1 h at room temperature with secondary antibody conjugated with peroxidase. Membrane-bound immune complexes were detected using Super ECL detection reagent on Amersham Imager 600(GE Healthcare).

### Cell viability assay

Cell viability was analyzed using the Cell Counting Kit-8 (CCK-8) (Dojindo). Cells at a density of 5 × 10^2^/well were seeded into 96-well plates and cultured in 100 μl of DMEM containing 10% FBS for 5 days. Ten microliter of CCK8 solution was added to each plate, and the cells were incubated for 3 h at 37 °C. The absorbance value (OD) of each well was measured at 450 nm.

### Colony formation assay

Cells were plated in 6-well culture plates. After incubation for 12 days at 37 °C, the cells were washed twice with PBS and stained with 0.1% crystal violet solution. The number of colonies was counted under a microscope.

### Migration and invasive assays

Cell motility was assessed by cell invasion and migration assays using Transwell chambers with or without Matrigel (BD, Biosciences). Cells in medium without FBS were seeded on Transwell chambers with or without Matrigel and incubated at 37 °C for 15 h. Medium containing 2% FBS was put in the lower chamber. The invasive cells attached to the lower surface of the membrane insert were fixed, stained using Giemsa and quantified.

### ChIP

ChIP assay was carried out using Chromatin Immunoprecipitation Kit (Sigma-Aldrich) according to the manufacturer’s protocol. IP was performed using specific antibodies, anti-RNA Pol II or normal IgG. Experiments were repeated three times independently.

### Promoter luciferase assay

Cells were transfected with reporter constructs containing the promoter together with expression vector or empty vector using Lipofectamine 3000 (Thermo Fisher Scientific). After 48 h, the luciferase activities of whole cell lysates were measured using the dual-luciferase reporter assay system (Promega). For all samples, the assays were repeated at least three times.

### Xenograft and metastasis assays

All animal studies were approved by the Institutional Animal Care and Use Committee of Xi’an Jiaotong University (Xi’an, China).Eight-week-old male nude mice Nude mice were housed five mice per cage in a specific pathogen-free room. HCC-LM3 (1.0 × 10^6^ cells/mouse) or MHCC97H (1.0 × 10^7^ cells/mouse) shMEIS2C/D and control cell lines were subcutaneously injected into the mice to form the xenograft model. The mice were randomly divided into indicated groups (5–10 mice/group) before inoculation, and double-blinded evaluation was performed. Mouse survival was analyzed using a survival analysis. The mice were sacrificed at endpoint, tumors and their lungs were removed. Tumor volume and weight were measured. To evaluate the potential of the cells to metastasize to livers, nude mice spleens were inoculated with shMEIS2C/D or control HCC-LM3 cells (8 × 10^5^), and the metastatic tumor colonies in the liver were measured 12 weeks later.

### Statistics analysis

Data analysis was performed using the SPSS software version 16. Survival of distinct subgroups of HCCs were compared by Kaplan-Meier and log-rank analyses. Results were presented as mean ± s.d. χ2-Test and two-tailed Student’s *t*-test were used to assess statistical significance. *P* < 0.05 was considered statistically significant.

## Results

### MEIS2C/D expression is significantly upregulated in HCC and it correlates with poor prognosis

**Fig. 1:**
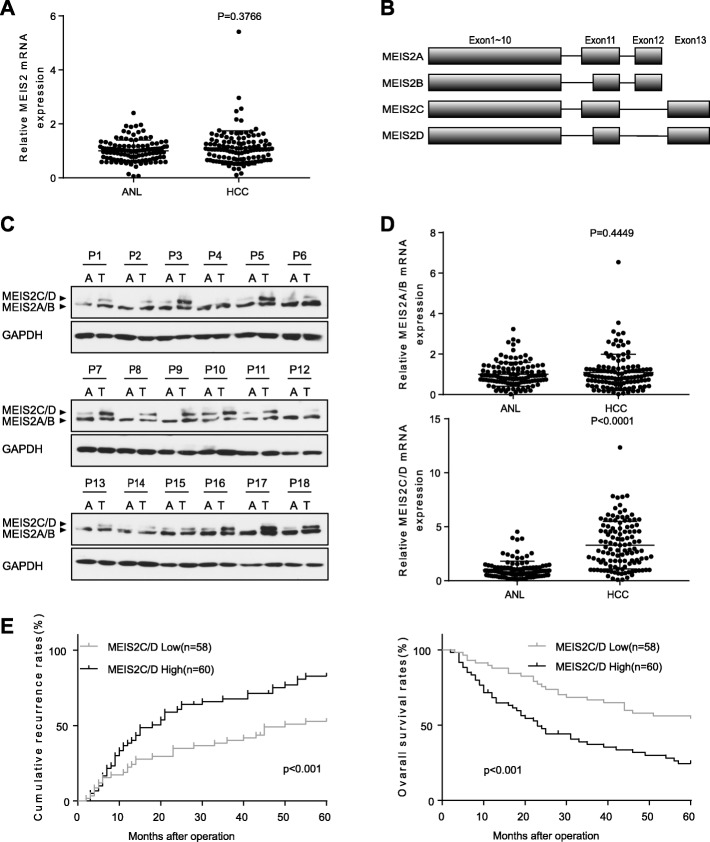
MEIS2C/D are overexpressed in hepatocellular carcinoma tissues and associated with poor prognosis. **a** Quantitative reverse transcriptase PCR (qRT-PCR) analysis showing the expression of MEIS2 in 118 pairs of HCC patient tissues compared adjacent noncancerous livers (ANL). **b** A schematic drawing of four major splice subtypes of MEIS2 mRNA. Alternative splicing generated two mainly group of MEIS2 isoforms with different C-terminal exon (exon12 vs. exon 13). **c** Western blot analysis of MEIS2 in 18 random HCC and paired ANL tissues. **d** Real-time PCR analysis of MEIS2A/B (exon12) and MEIS2C/D (exon13) expression in HCC and matched ANL. **e** Prognostic significance assessed by Kaplan–Meier estimates method and log-rank test. Comparison of the time to recurrence rate and overall survival by MESI2C/D expression

To determine whether MEIS2 has important roles in liver cancer, we compared the expression of MEIS2 mRNA and protein in a cohort of 118 human HCCs and their paired adjacent noncancerous liver (ANL) samples. The results showed that MEIS2 mRNA was not significantly changed in HCC tissues when compared with ANL (Fig. 1a). Due to alternative splicing, there are four MEIS2 isoforms with different C-termini. Skipping of exon 12 results in a longer C-terminus, present in isoforms MEIS2C/D, whereas inclusion of exon 12 introduces an early stop codon and generates a distinct and shorter C-terminus, termed MEIS2A/B (Fig. 1b). Strikingly, when we investigated the protein level of different MEIS2 isoforms in 18 random HCC and matched ANL tissues, we found that MEIS2C/D is overexpressed in these HCC tissues, whereas MEIS2A/B is not significantly changed (Fig. 1c). Similarly, there is no significant difference in the expression status of MEIS2A/B between HCC and ANL tissues, whereas the mRNA level of MEIS2C/D was significantly increased in HCC tissues (Fig. 1d). Next, we divided HCC patients into low MEIS2C/D group and high MEIS2C/D group based on their relative median mRNA expression level of MEIS2C/D and compared 12 widely recognized clinicopathological parameters in 118 HCC specimens. Statistical analysis indicated that higher MEIS2C/D expression level was not only significantly associated with more advanced tumor stage and differentiation, but also significantly associated with tumor size and the presence of microvascular invasion (Additional file 1: Table S1). These results indicated that MEIS2C/D is associated with HCC growth and metastasis. Furthermore, Kaplan-Meier’s survival curves showed that the HCCs with a higher MEIS2C/D expression had higher cumulative recurrence rates (CRR) after hepatectomy and worse overall survival (OS) (Fig. 1e). These results indicated that MEIS2C/D might promote tumor progression of HCC and that it could serve as a helpful prognostic marker for HCC patients after hepatectomy.

### Knockdown of MEIS2C/D inhibited proliferation and metastasis of HCC cells in vitro

**Fig. 2:**
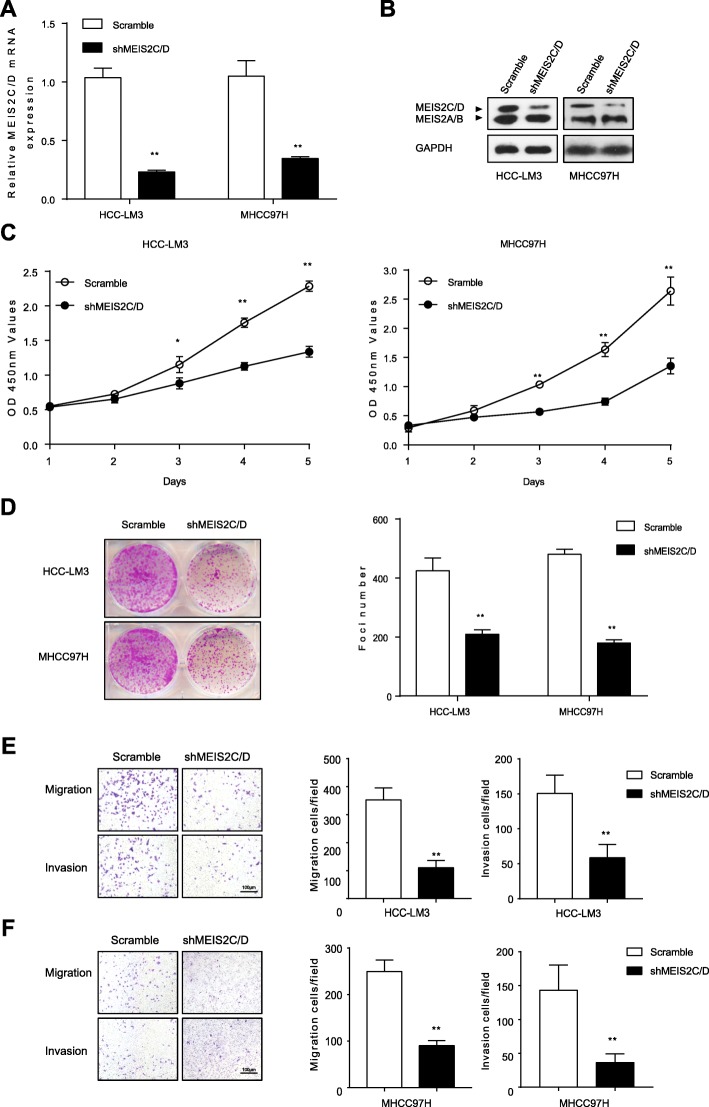
Knockdown MEIS2C/D inhibited growth and metastasis of HCC cells in vitro. **a, b** Expression of *MEIS2C/D* were measured by qRT-PCR and western blot in HCC-LM3 and MHCC97H cells treated with shMEIS2C/D targeting MEIS2C/D (exon 13) or Scramble. **c**, **d** The in vitro proliferation function of MEIS2C/D was measured by the CCK-8 Kit and colony-forming assay. The cells were seeded in 6-well plates. **e**, **f** Migration and invasion were measured by Transwell assay coated with or without Matrigel in the indicated stable HCC cell lines. HCC cells were seeded in 12-well plates. Representative images are shown (scalebar 100 μm). Experiments were repeated three times with similar results, and error bars represent the mean ± SEM, *P < 0.05, **P < 0.001

To further define the biological role of MEIS2C/D in HCC, we designed short hairpin RNA and siRNA to knockdown MEIS2C/D expression (Fig. 2a, b and Additional file 1: Figure S1). MEIS2C/D mRNA and protein expression in HCC cell lines (HCC-LM3 and MHCC97H) were modulated by transfection with shMEIS2. Afterwards, we examined the effect of MEIS2C/D knockdown on HCC cells growth in vitro. By using CCK-8 and colony formation assays (Fig. 2c, d), we found that knockdown of MEIS2C/D markedly inhibited proliferation in HCC cells. Furthermore, matrigel-uncoated (for migration) or –coated (for invasion) Transwell assays showed that HCCs migration and invasion were effectively suppressed by MEIS2C/D knockdown (Fig. 2e, f).

### MEIS2C/D suppression impaired HCCs growth and metastasis in vivo

**Fig. 3:**
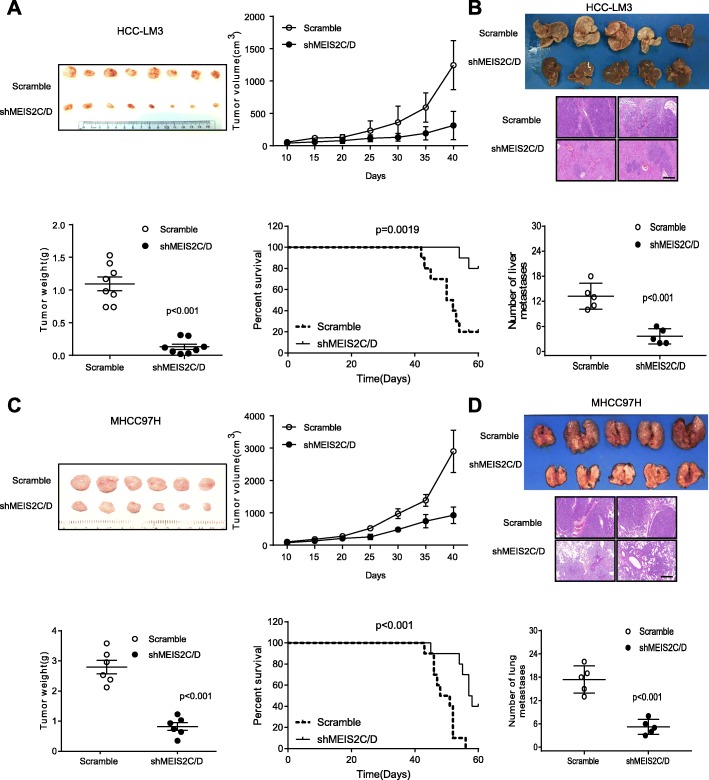
Knockdown MEIS2C/D suppressed malignant behaviors of HCC cells in vivo. **a** Representative image showing xenografts in nude mice were established by subcutaneous injection of shMEIS2C/D and control HCC-LM3 cell lines (*n* = 8). Comparisons of overall survival curves in mice (*n* = 10). *P* values were calculated using the Log-rank test. **b** Representative image showing visible intrahepatic metastases in the HCC-LM3-shMEIS2C/D and control groups (*n* = 5). **c** MHCC97H-shMEIS2C/D and control cells were injected subcutaneously in nude mice and tumor volume was evaluated after injection (*n* = 6). Comparisons of overall survival curves in mice injection of MHCC97H-shMEIS2C/D and control groups (*n* = 10). **d** Representative image and H&E stain showing spontaneous lung metastasis in matched subcutaneous nude mice model (*n* = 5)

To translate the in vitro findings in vivo, we first established xenograft tumor in nude mice by subcutaneously injecting shMEIS2C/D and control stable HCC cell lines. After 6 weeks of tumor inoculation, shMEIS2C/D cell-derived HCC-LM3 and MHCC97H tumors at the subcutaneous implantation sites were smaller and grew more slowly than those in the scramble group (Fig. 3a, c). Next, we inoculated shMEIS2C/D HCC-LM3 and control cells into mouse spleens to explore their hepatic metastasis potentials in nude mice. After 12 weeks, we measured metastatic tumor colonies in liver, and found that the group inoculated with HCC-LM3 shMEIS2C/D cells showed fewer and smaller xenografts in the liver than the control group (Fig. 3b). In addition, the pulmonary metastasis rates in nude mice with tumors derived from shMEIS2C/D-MHCC97H cells were also significantly lower than the corresponding control group (Fig. 3d). Collectively, these findings suggested that MEIS2C/D is capable of promoting HCC aggressive and metastatic phenotype in vivo.

### MEIS2D promotes miR-1307-3P expression in HCC

**Fig. 4:**
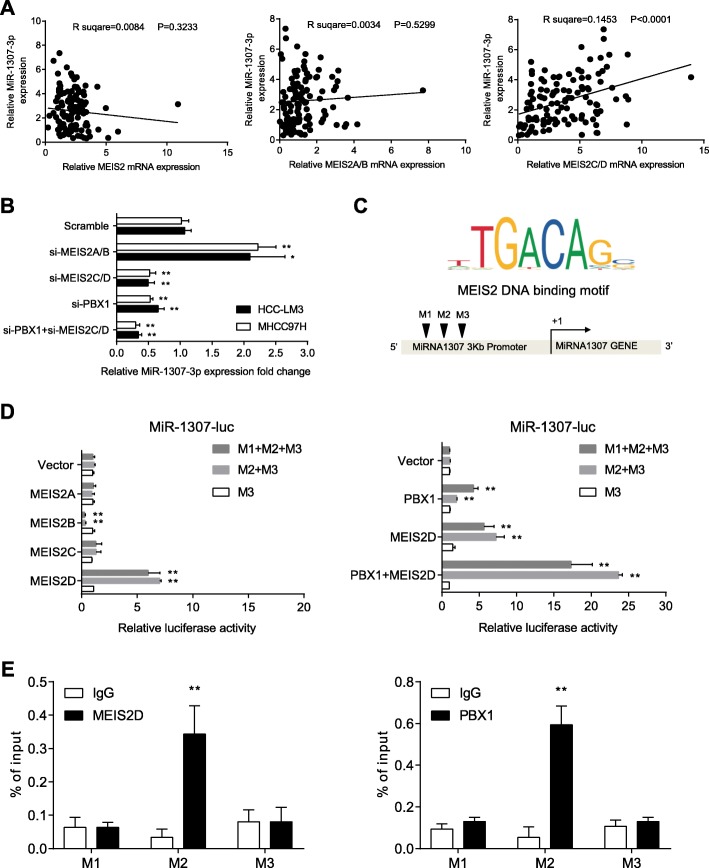
MEIS2 regulated miR-1307-3p expression. **a** The correlation between the mRNA expression of MEIS2 and miR-1307-3p in HCC patient cohort. Pearson’s correlation analysis was used. **b** miR-1307-3p expression were detected by qRT-PCR after transfection of indicated siRNA in HCC cell lines. **c** The predicted putative MEIS2 binding site on miR-1307 promoter by using JASPAR (jaspar.genereg.net). **d** Dual luciferase reporter assays of various recombinant miR-1307 promoter vectors were co-transfect with different isoforms of MEIS2 and its coactivator PBX1 in HEK293T cells. Relative luciferase activity was determined by normalizing the activity of Firefly against Renilla luciferase. **e** ChIP assay was performed to examine the association of MEIS2D and PBX1 with the potential MEIS2 binding site in different miR-1307 promoter regions (3 KB), as determined by qRT-PCR

Although we have confirmed that MEIS2C/D could promote the progression and metastasis of HCC in vitro and in vivo, the underlying molecular mechanism remains to be explored. Given the findings that miR-1307-3p has been implicated in tumorigenesis [18, 19], we hypothesized that miR-1307-3p may as a potential target of MEIS2. Although neither MEIS2 nor MEIS2A/B correlated with miR-1307-3p expression in HCC, MEIS2C/D positively correlated with the expression of miR-1307-3P (Fig. 4a). MEIS2 and PBX1 are member of the TALE superfamily of homeodomains, and PBX1 can also recognize and co-activate MEIS2 near the MEIS2 binding motif sequence. When we used siRNA to knockdown the levels of MEIS2C/D or PBX1, the expression of miR1307-3p drastically decreased (Fig. 4b). Moreover, by sequence analysis, we found that there are three putative MEIS2 binding sites in the promoter of miR-1307 gene (Fig. 4c). To test whether MEIS2 could activate the expression of miR-1307 through these three loci, we constructed a series of reporters containing these three locus sequences and co-transfected HEK293T cells with expression plasmids of four MEIS2 isoforms. Consistently, the luciferase activity assay indicated that, when compared with the other isoforms, MEIS2D could significantly activate the activity of M2 + M3 and M1 + M2 + M3 reporter (Fig. 4d, Left panel). Furthermore, MEIS2D and PBX1 could synergistically enhance the activity of miR-1307 promoter (Fig. 4d, Right panel). To further determine the binding site of MEIS2D and PBX1 on miR-1307 promoter, we designed primers at the M1, M2 and M3 loci of miR-1307 promoter and performed ChIP assay. Our data confirmed that both MEIS2D and PBX1 could bind to M2 site directly (Fig. 4e and Additional file 1: Figure S2A). Notably, MEIS2A/B knock down could suppress miR-1307-3p expression and MEIS2B overexpression inhibited reporter of miR-1307 promoter (Fig. 4d, Additional file 1: Figure S2B and S2C). Therefore, we theorized that the M2 site of miR-1307 promoter mediates the binding and transcriptional activation of MEIS2D and PBX1 complex.

### MEIS2C/D suppression inhibited nuclear accumulation of YAP by miR-1307-3p/LATS1 axis

**Fig. 5:**
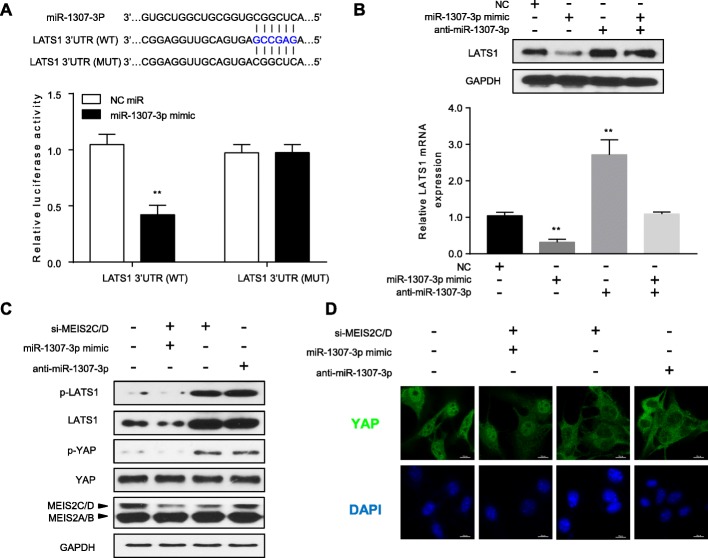
Knockdown MEIS2C/D inhibited nuclear accumulation of YAP by miR-1307-3p/LATS1/YAP axis. **a** Diagrams indicate putative miR-1307-3p binding sites and corresponding mutant sites of LATS1 mRNA. LATS1 wild type mRNAs (WT) and corresponding mutant sequences (MUT), which was cloned in luciferase reporter and co-transfection of either negative control or miR-1307-3p mimic in HEK293T cells. **b** LATS1 protein and mRNA levels were measured by western blot and qRT-PCR in HEK293T cells transfected with miR-1307-3p mimic, anti-miR-1307-3p or negative control (NC) miRNA. **c** HCC-LM3 cells were transfected with anti-miR-1307-3p, miR-1307-3p mimic, MEIS2C/D siRNA or controls. Then, Immunoblotting of cell lysates was performed to examine phosphorylation of LATS1 and YAP. **d** HCC-LM3 cells were transfected with indicated siRNA, miRNA and inhibitor. YAP intracellular localization investigated by immunofluorescence staining

Alterations in the components of the Hippo signaling pathway are reported to be associated with tumor progression in HCC [25, 26]. The central axis of the Hippo pathway is a kinase cascade that includes MST1/2 and LATS1/2. Interestingly, our results showed that 3′ untranslated region (UTR) of LATS1 mRNA had putative binding sequence for miR-1307-3p (Fig. 5a, Upper panel from www.targetscan.org), indicating that LATS1 might be a target gene of miR-1307-3p. In order to confirm this, we transfected HEK293T cells with miR-1307-3p mimics and detected the luciferase activity of LATS1 3′ UTR reporter. We found that the luciferase activity of LATS1 mRNA reporter was significantly inhibited when miR-1307-3p was overexpressed. By contrast, when we mutated the predicted binding site sequence for miR-1307-3p, the expression of LATS1 was no longer affected by miR-1307-3p (Fig. 5a, lower panel). Moreover, qRT-PCR and western blot assays revealed that miR-1307-3p overexpression dramatically reduced LATS1 levels in HEK293T cells (Fig. 5b). As the regulatory molecules of YAP in Hippo pathway, LATS1 can reduce the level of phosphorylation of YAP. Based on this, si-MEIS2C/D, miR-1307-3p mimic or anti-miR-1307-3p were used to transfect HCC-LM3 cells, and LATS1 and YAP were detected by Western blotting. When the expression of MEIS2C/D or miR-1307-3p was inhibited, the protein levels of LATS1 and phosphorylated LATS1 increased significantly, while YAP, the substrate of LATS1, was degraded by LATS1 phosphorylation, resulting in an increased in phosphorylation level (Fig. 5c). We also performed immunofluorescence assay and confirmed that the nuclear localization of YAP was weakened by si-MEIS2C/D, miR-1307-3p mimic or anti-miR-1307-3p transfections (Fig. 5d and Additional file 1: Figure S3B).

### MEIS2C and MEIS2D collaborated with CDC73 in differential modes

**Fig. 6:**
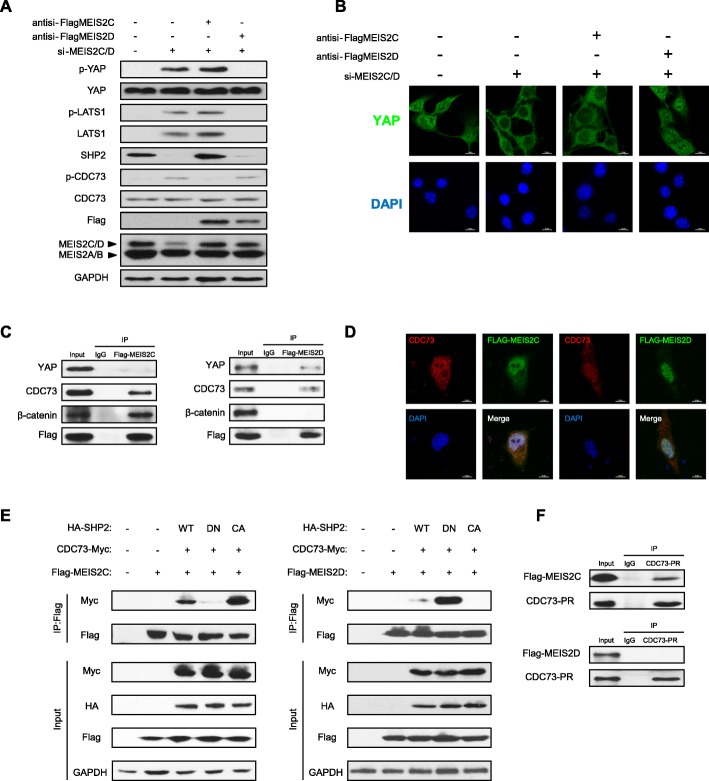
MEIS2C/D interacted with β-catenin and YAP in different modes. **a** The rescue MEIS2C or MEIS2D cDNA clone, which is resistant to siRNA interference, were used to rescue the MEIS2C/D expression after MEIS2C/D knockdown in HCC-LM3 cells. The level of phospho-LATS1, phospho-YAP, phospho-CDC73 and total LATS1, YAP, CDC73 and SHP2 were detected in MEIS2C and MEIS2D rescued HCC-LM3 cells, respectively. **b** Immunofluorescence staining for DAPI (Blue), YAP (Green) in HCC-LM3 cells. **c** Lysates were sequentially immune-precipitated (IP) from HCC-LM3 cells transfected with Flag-MEIS2C (left) or Flag-MEIS2D (right) and immunoblotted by CDC73, β-catenin and YAP antibody. **d** Immunofluorescence images showing dual staining for CDC73 (Red) with Flag-MEIS2C or Flag-MEIS2D (Green) in HCC-LM3C cells. Merged picture with sites of co-localization in yellow are shown. **e** SHP2 constructs [wildtype (WT), dominant negative (DN, C463S mutant), and constitutively active (CA, E76K mutant)] and CDC73-Myc co-transfected with Flag-MEIS2C or Flag-MEIS2D in HEK293T cells. MEIS2 were immune-precipitated from whole cell lysates and probed with Myc antibody to check the interaction with CDC73. **f** Phosphorylation-resistant (PR) CDC73-PR-Myc mutant (CDC73-Y290/293F/315F-mutant) vector was transfected together with either Flag-MEIS2C or Flag-MEIS2D vector into HEK293T cells. Anti-Myc immune-precipitates were analyzed by immunoblotting with anti-Myc and anti-Flag antibodies

Since MEIS2D, not MEIS2C, could promote miR-1307 transcription, we speculate that MEIS2C/D may engage in different molecular programs in the progression of HCC. In order to distinguish the molecular function of MEIS2C and MEIS2D, firstly we suppressed the expression of endogenous MEIS2C/D in HCC-LM3 and MHCC97H cell lines, and then transfected cells with anti-si-FlagMEIS2C and anti-si-FlagMEIS2D plasmids, which are resistant to the siRNA of MEIS2C/D (Fig. 6a and Additional file 1: Figure S4B). We confirmed that MEIS2D suppressed YAP phosphorylation through LATS1/miR-1307-3p axis. Moreover, protein level of SHP2 was decreased in anti-si-FlagMEIS2D transfected group. Correspondently, the results of immunofluorescence confirmed the nuclear translocation of YAP in the MEIS2D rescued group (Fig. 6b and Additional file 1: Figure S4A). Interestingly, Subsequent co-immunoprecipitation experiment showed that MEIS2D interacted with YAP and CDC73, while MEIS2C could bind to β-catenin and CDC73 (Fig. 6c). Furthermore, immunofluorescence assay showed that the nuclear localization of CDC73 and MEIS2C/D was similar (Fig. 6d).

It is noteworthy that CDC73 is involved in different transcription complex according to its phosphorylation status [24, 27]. To address whether the phosphorylation status of CDC73 could determine its association with either MEIS2C or MEIS2D, we co-transfected HEK293T cells with wild type (WT), dominant negative (DN, C463S-SHP2), and constitutive active (CA, E76K-SHP2) SHP2 together with CDC73 and MEIS2C/2D. By using co-immunoprecipitation, we found that dominant negative SHP2 exogenous expression significantly suppressed the interacting between CDC73 and MEIS2C, and the interaction could be further enhanced by constitutive active SHP2 (Fig. 6e left). Conversely, dominant negative SHP2 facilitated the interaction between CDC73 and MEIS2D, and this model can also be attenuated by constitutive active SHP2 (Fig. 6e right). Previous study reported that the phosphorylation-resistant (PR) CDC73 mutant could inhibit the interaction between CDC73 and other co-activators [27]. To explore the interaction between CDC73 and MEIS2C/D, we expressed CDC73PR mutant to immunoprecipitated Flag-MEIS2C or Flag-MEIS2D in HEK 293 T cells. The results showed that CDC73PR mutant could bind to MEIS2C as opposed to MEIS2D (Fig. 6e right). These observations collectively support that MEIS2C and MEIS2D involve in different transcription complex: MEIS2C binds to β-catenin and dephosphorylated CDC73. In contrast, MEIS2D interacts with YAP and phosphorylated CDC73.

### MEIS2C and MEIS2D promoted oncogene transcription in human liver cancer

**Fig. 7:**
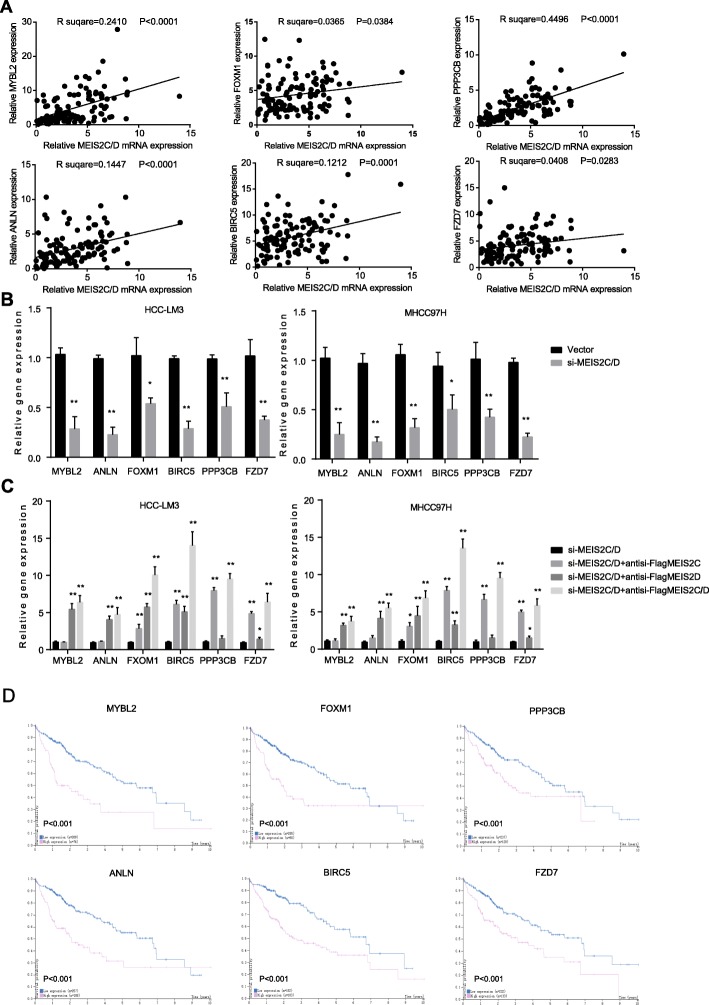
MEIS2C/D promoted HCC related oncogene expression. **a** qRT-PCR analysis showed the RNA expression of MEIS2C/D was associated with MYBL2, ANLN, FOXM1, BIRC5, PPP3CB and FZD7 expression were investigated in HCC cohort. **b**, **c** Gene expression were investigated by qRT-PCR transfected with siMEIS2C/D or rescued expression with indicated antisi-FlagMEIS2 vectors in HCC-LM3 and MHCC97H cells. **d** Overall survival for patients with high and low MEIS2C/D related gene expression in HCC cohorts (https://www.proteinatlas.org)

After we established the link between β-catenin, YAP, CDC73, and MEIS2C/D, we sorted to determine the downstream target genes of MEIS2C/D in HCC. From database mining and screening [28], we identified six MEIS2C/D downstream HCC-related genes, including MYBL2, ANLN, FOXM1, BIRC5, PPP3CB and FZD7 (Fig. 7a and Additional file 1: Figure S5). Importantly, knockdown of MEIS2C/D suppressed these genes expression in HCC cell lines (Fig. 7b). MYBL2, ANLN and FOXM1 are known as target genes of YAP [29, 30], while BIRC5, PPP3CB and FZD7 are related to βcatenin signaling [31]. To further explorer the transcriptional activation of these two MEIS2 isoforms, we suppressed MEIS2C/D expression, and then ectopic expressed MEIS2C and MEIS2D in HCC cells. Of note, MEIS2C and MEIS2D could either promote gene transcription synergistically or non-synergistically (Fig. 7c). In support of the putative oncogenic activities of MEIS2C/D, higher expression of these genes correlated with poorer prognosis in HCC patients (Fig. 7d, from www.proteinatlas.org). Herein, we suggest that the activation of these genes exerts the carcinogenic effect of MEIS2C/D and eventually contributes to the development of HCC.

## Discussion

Due to the molecular mechanism of HCC pathogenesis remain unclear, many HCC patients are diagnosed as metastasis and advanced stage, therefore missed the opportunity for surgical treatment with good prognosis. Deciphering the molecular mechanisms of carcinogenesis and metastasis is crucial for the development of new therapeutic strategies in HCC. Most recent research indicated that MEIS2D has the effect of promoting cancer growth in Neuroblastoma [9, 15]. However, MEIS2 is down-regulated in advanced prostate cancer [32]. In this study, we report that the expression of MEIS2C/D was significantly up-regulated and correlated with poor prognosis which may serve as a promising therapeutic target in personalized treatment of HCC. Based on the above results, we suppose that MEIS2C/D have significant impact on tumor growth and metastasis in liver cancer cells. After the expression of MEIS2C/D was attenuated in HCC cell lines, proliferation and metastasis of these cells were suppressed significantly in vitro, and the rate of subcutaneous tumorigenesis and metastasis in mice were also significantly decreased in vivo.

MiR-1307-3p is a member of the miR-1307 sub-family and plays an important role in breast cancer, colorectal cancer and ovarian cancer, but the effect in HCC remain unclear [14, 33, 34]. Moreover, accumulating studies demonstrates that miR-1307-3p also relate to chemotherapy resistant ovarian cancer [35, 36]. Considering the transcriptional property of MEIS2 family and the correlation expression between MEIS2C/D and miR-1307-3p in HCC patients, our studies demonstrate that miR-1307-3p is one of the key components of the MEIS2D circuit, whereby the expression of miR-1307-3p is driven by MEIS2D in HCC cells. MEIS2D and its synergistic molecule, PBX1, could co-activate the expression of miR-1307. Moreover, this proposal ensues that miR-1307-3p may act on its target gene and then help MEIS2D to further activate the signaling pathway in HCC progression. After analysis the potential target of miR-1307-3p, we identified that LATS1 as a functional target of miR-1307-3p. Consequently, LATS1 suppression decreased the phosphorylation of YAP and promoted the nucleation of YAP. In addition, YAP interacted with MEIS2D and phosphorylated CDC73, then promoted transcription of downstream gene. In miRNA biogenesis, miR-1307-5p and miR-1307-3p mature miRNA can be excised from the same pre-miRNA. The expression of miRNA is controlled at transcriptional and post-transcriptional levels. We identified that MEIS2D promoted the transcription of miR-1307, but post-transcriptional regulation of miR-1307-3p remains elusive.

Although MEIS2C and MEIS2D play similar proliferative role in tumor progression [9], it is important to distinguish the pathological function between these two isoforms. Our studies demonstrate that CDC73 interacts with either MEIS2C/β-catenin or MEIS2D/YAP in a trimeric complex. Notably, it has been reported that WNT, NOTCH and YAP are inversely regulated by CDC73 according to its tyrosine phosphorylation status, and coordinates activation of genes targeted by these complex [24, 27]. Correspondingly, we confirmed that MEIS2C and MEIS2D exclusive interacted with CDC73 also depending on its tyrosine phosphorylation status.

SHP2, also known as PTPN11, has been elucidated its crucial role in liver cancer stem cell expansion [23]. Reduction of SHP2 activity suppresses cancer cell growth and is a potential target of cancer therapy [37]. However, it has been reported that SHP2 localization regulated by YAP is different in vitro and in vivo in hepatocellular carcinogenesis [38, 39]. Of note, we found that SHP2 decreased by MEIS2C/D knockdown (Additional file 1: Figure S3A). What is noteworthy is that the rescued MEIS2C may increase the protein level of SHP2 (Fig. 6a), which in turn enhances its interaction with CDC73 which act as an important substrate of SHP2. Although MESI2C and MEIS2D are competitive binding to CDC73, the present works reveal that crosstalk also exist between these two isoforms such as co-activation of FXOM1 and BIRC5 transcription.

Our clinical investigation revealed that the protein level of MEIS2A/B was generally higher than that of MEIS2C/D. So we speculate that MEIS2A/B also performs important biological functions in HCC. Considerable evidences indicate that MEIS2A inhibits the development of neuroblastoma [15], and also our results confirmed that MEIS2B overexpression inhibited the transcription of miR-1307. However, our studies indicate that protein and mRNA level of MEIS2A/B are no difference between HCC and ANL tissues. Therefore the function of MESIA/B remains to be further explored in HCC patients.

Taken together, our study shows that MEIS2D promotes the development of HCC by miR-1307-3p/LATS1/YAP circuit. On the other hand, MEIS2C bind to dephosphorylated CDC73 and activate β-catenin in a trimeric complex. Although these data imply that MEIS2C/D might be a potential biomarker for HCC treatment, further clinical investigation and more data analysis are warranted to establish this point. The significance of MEIS2C/D in HCC personalized medicine is deserved to be further investigation.

## Conclusions

Given Hepatocellular Carcinoma (HCC) is the third leading cause of cancer death worldwide, defining the regulatory mechanism is essential to decipher the pathogenesis of HCC. Recent efforts have identified MEIS2 as one of the key transcription factors in human cancers. However, the regulatory mechanisms of MEIS2 activity in HCC remain obscure. Herein, we describe that two of MEIS2 alternatively spliced isoforms, MEIS2C and MEIS2D, expression dramatically increased in HCC patient, and negatively correlated with the prognosis of HCC patients. Using an in vivo and in vitro approach, we show that MEIS2C and MEIS2D promote the proliferation and metastasis of hepatoma cells via Hippo/YAP and Wnt/β-catenin signaling. This provides further insights into the complex regulatory network of MEIS2 and further support for developments of MEIS2 isoforms–specific inhibitors for HCC treatment.

## Supplementary information

**Additional file 1 **: **Figure S1.** (A) siMEIS2 efficiency measured by western blot in HEK293T cells. (B) Relative MEIS2C/D mRNA levels were measured by q RT-PCR in different cell lines. **Figure S2.** (A) Three predicted MEIS2 binding sites detected by ChIP in HEK293T cells. Input DNA were used as positive controls. Immuno-precipitated DNA by anti-IgG antibody was used as negative controls. PCR was used to find out the MEIS2D and PBX1 binding site for miR-1307 gene. (B) Results of ChIP of M2 sites DNA fragments performed with Flag-MEIS2A/B/C. (C) Results of ChIP of three predicted MEIS2 binding sites DNA fragments performed with Flag-MEIS2B. **Figure S3.** (A) HCC-LM3 cells were transfected with indicate siRNA, miRNA or controls. Then, immunoblotting was performed to examine SHP2. (B) YAP intracellular distribution detected by immunofluorescence staining. (C, D) HCC-LM3 cells were transfected with indicated plasmids, siRNAs and controls. Then, Immunoblotting and immunofluorescence staining were performed to examine LATS1 and YAP expression and localization. **Figure S4.** (A) MHCC97H cells were transfected with indicated siRNA and vectors. YAP localization investigated by immunofluorescence staining. (B) Lysates were detected by indicated antibody. HCC-LM3 and MHCC97H cells were transfected with siMEIS2 and indicated rescue vectors. Then, proliferation function of MEIS2C or MEIS2D was measured by CCK-8 Kit(C). Relative YAP and β-catenin mRNA levels were measured by q RT-PCR after 48 h (D). **Figure S5.** RNA expression correlation between MEIS2A/B and genes were investigated in HCC patients. **Table S1.** Correlations between clinic pathological features and relative MEIS2C/D expression. **Table S2.** Predicted putative MEIS2 binding site sequence and position on miR-1307 Promoter (3 KB) by using JASPAR (jaspar.genereg.net)

## Data Availability

All data generated during this study are included in this published article.

## Abbreviations

ANL: Adjacent noncancerous liver
CDC73: Cell division cycle 73, Parafibromin
ChIP: Chromatin immunoprecipitation
CRR: Cumulative recurrence rate
H&E stain: Hematoxylin and eosin stain
HCC: Hepatocellular carcinoma
MEIS2: Meis homeobox 2
MEIS2A/B: MEISI2A and MEIS2B
MEIS2C/D: MEISI2C and MEIS2D
OS: Overall survival
qRT-PCR: Quantitative reverse transcriptase PCR
SHP2: Src homology phosphotyrosine phosphatase 2
TALE: Three amino acid loop extension
YAP: Yes-associated protein
