# Supplementary material for: MEIS2C and MEIS2D promote tumor progression via Wnt/β-catenin and hippo/YAP signaling in hepatocellular carcinoma
Source: J Exp Clin Cancer Res. 2019 Oct 17;38:417. doi: 10.1186/s13046-019-1417-3 (PMC6796342; doi:10.1186/s13046-019-1417-3)
Supplement: Supplementary file 1 — Additional file 1 : Figure S1. (A) siMEIS2 efficiency measured by western blot in HEK293T cells. (B) Relative MEIS2C/D mRNA levels were measured by q RT-PCR in different cell lines. Figure S2. (A) Three predicted MEIS2 binding sites detected by ChIP in HEK293T cells. Input DNA were used as positive controls. Immuno-precipitated DNA by anti-IgG antibody was used as negative controls. PCR was used to find out the MEIS2D and PBX1 binding site for miR-1307 gene. (B) Results of ChIP of M2 sites DNA fragments performed with Flag-MEIS2A/B/C. (C) Results of ChIP of three predicted MEIS2 binding sites DNA fragments performed with Flag-MEIS2B. Figure S3. (A) HCC-LM3 cells were transfected with indicate siRNA, miRNA or controls. Then, immunoblotting was performed to examine SHP2. (B) YAP intracellular distribution detected by immunofluorescence staining. (C, D) HCC-LM3 cells were transfected with indicated plasmids, siRNAs and controls. Then, Immunoblotting and immunofluorescence staining were performed to examine LATS1 and YAP expression and localization. Figure S4. (A) MHCC97H cells were transfected with indicated siRNA and vectors. YAP localization investigated by immunofluorescence staining. (B) Lysates were detected by indicated antibody. HCC-LM3 and MHCC97H cells were transfected with siMEIS2 and indicated rescue vectors. Then, proliferation function of MEIS2C or MEIS2D was measured by CCK-8 Kit(C). Relative YAP and β-catenin mRNA levels were measured by q RT-PCR after 48 h (D). Figure S5. RNA expression correlation between MEIS2A/B and genes were investigated in HCC patients. Table S1. Correlations between clinic pathological features and relative MEIS2C/D expression. Table S2. Predicted putative MEIS2 binding site sequence and position on miR-1307 Promoter (3 KB) by using JASPAR (jaspar.genereg.net) [file 13046_2019_1417_MOESM1_ESM.pdf]

Figure S1

A

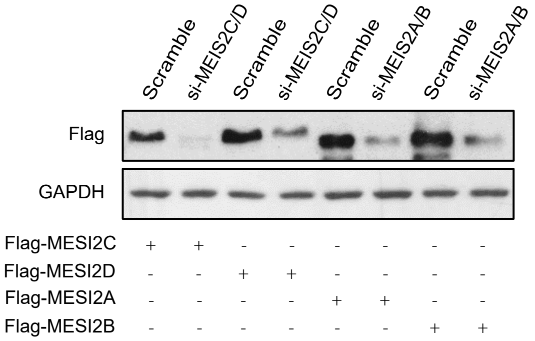

Scramble
si-MEIS2C/D
Scramble
si-MEIS2C/D
Scramble
si-MEIS2A/B
Scramble
si-MEIS2A/B
Flag
GAPDH
Flag-MESI2C + + - - - - - -
Flag-MESI2D - - + + - - - -
Flag-MESI2A - - - - + + - -
Flag-MESI2B - - - - - - + +

B

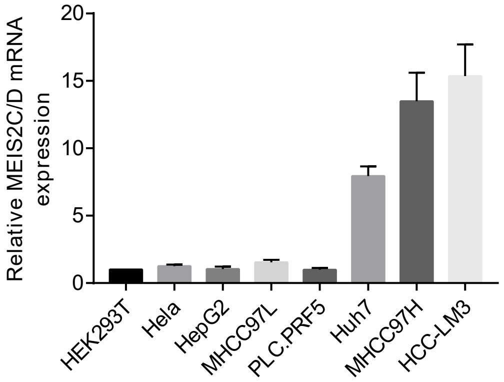

Relative MEIS2C/D mRNA expression
20
15
10
5
0
HEK293T
Hela
HepG2
MHCC97L
PLC.PRF5
Huh7
MHCC97H
HCC-LM3

Figure S2

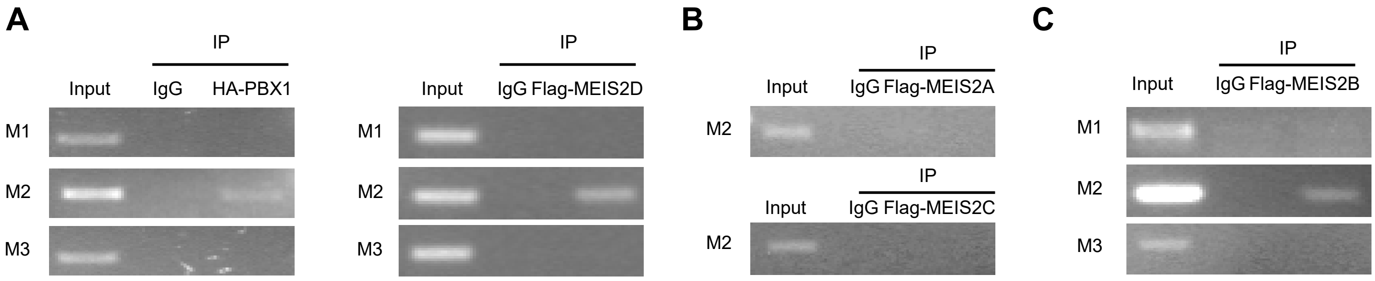
A
IP
Input
IgG
HA-PBX1
M1
M2
M3
IP
Input
IgG Flag-MEIS2D
M1
M2
M3
B
IP
Input
IgG Flag-MEIS2A
M2
IP
Input
IgG Flag-MEIS2C
M2
C
IP
Input
IgG Flag-MEIS2B
M1
M2
M3

Figure S3

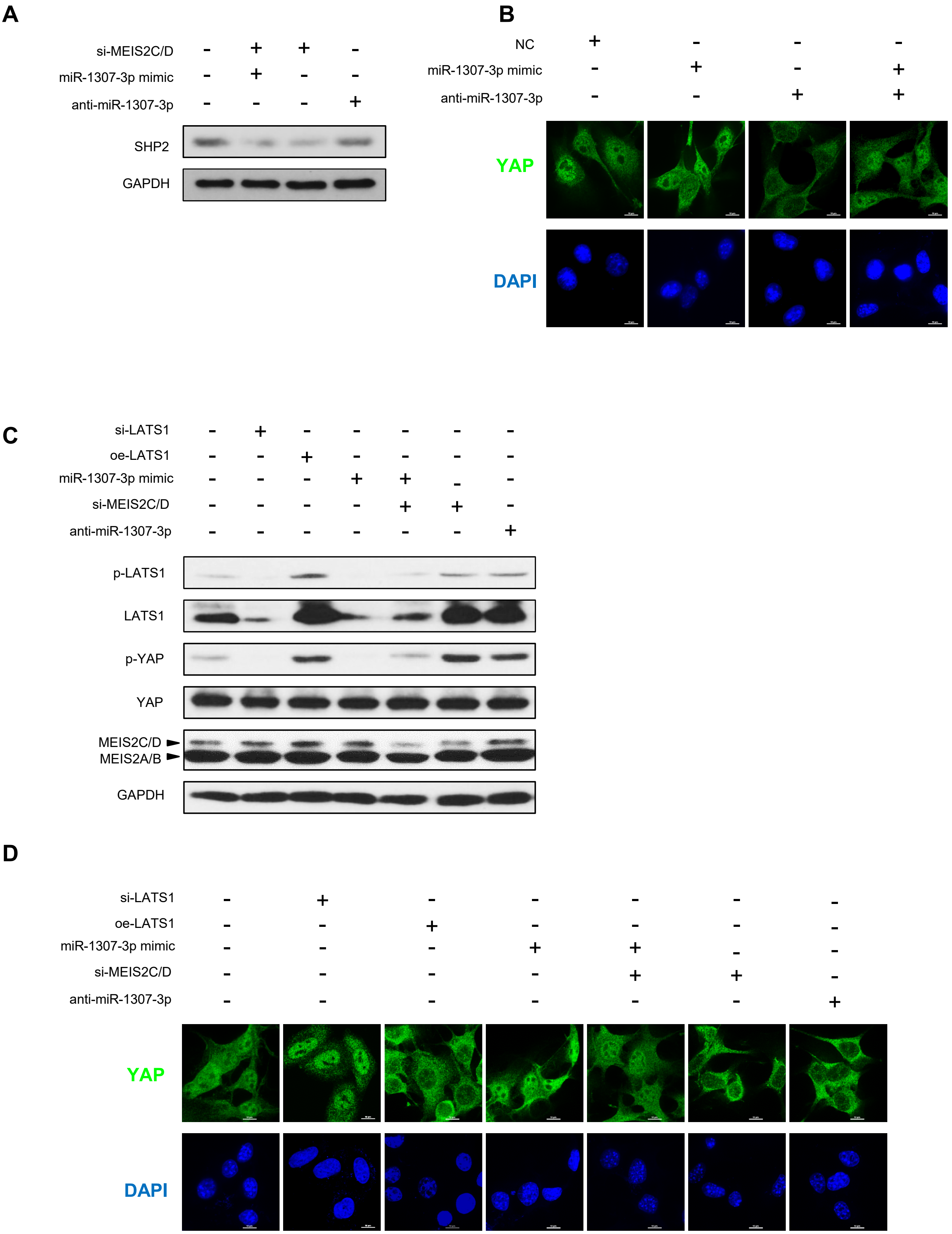
A
si-MEIS2C/D
miR-1307-3p mimic
anti-miR-1307-3p
SHP2
GAPDH
B
NC
miR-1307-3p mimic
anti-miR-1307-3p
YAP
DAPI
C
si-LATS1
oe-LATS1
miR-1307-3p mimic
si-MEIS2C/D
anti-miR-1307-3p
p-LATS1
LATS1
p-YAP
YAP
MEIS2C/D
MEIS2A/B
GAPDH
D
si-LATS1
oe-LATS1
miR-1307-3p mimic
si-MEIS2C/D
anti-miR-1307-3p
YAP
DAPI

Figure S4

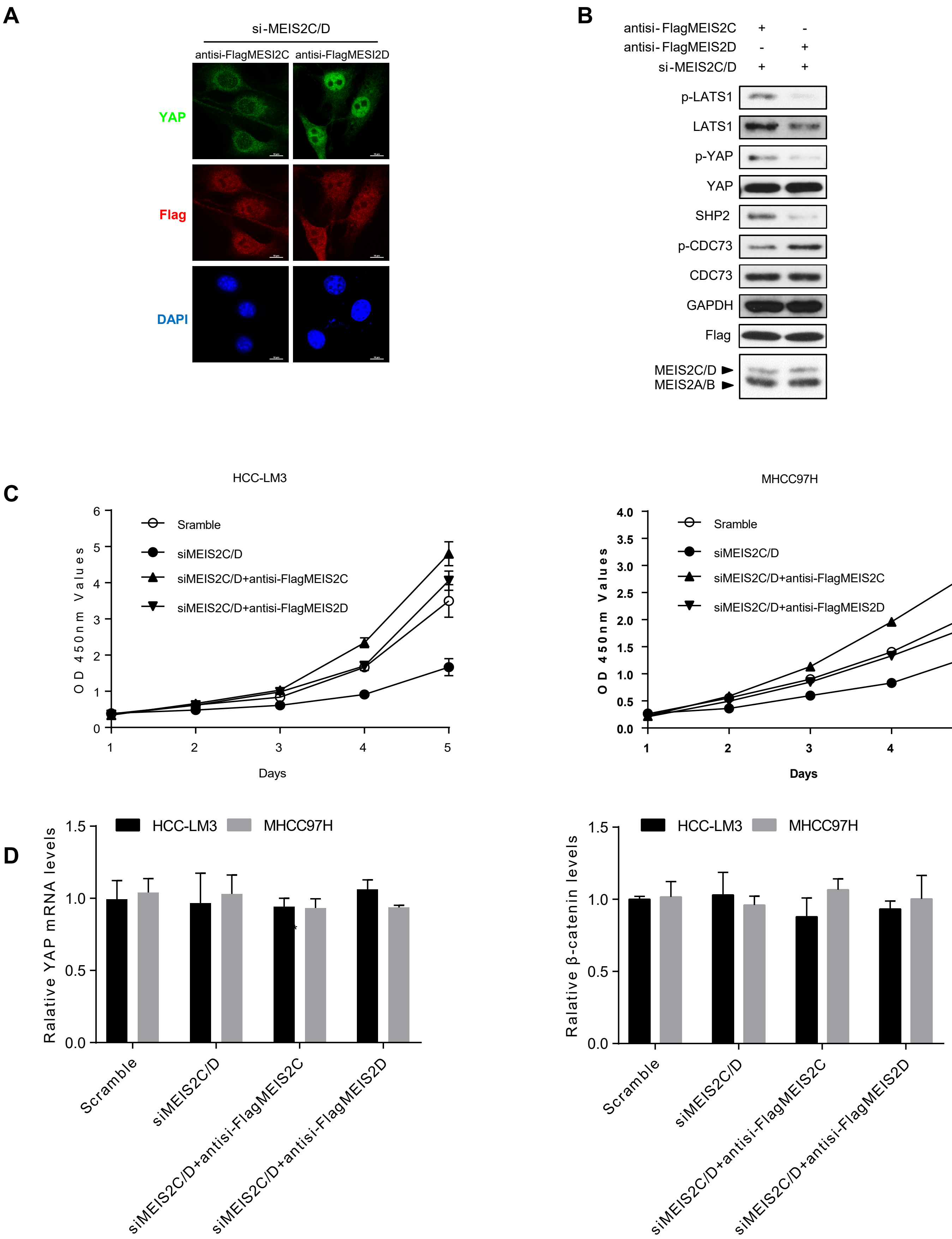
A
si-MEIS2C/D
antisi-FlagMESI2C
antisi-FlagMESI2D
YAP
Flag
DAPI
B
antisi-FlagMEIS2C + -
antisi-FlagMEIS2D - +
si-MEIS2C/D + +
p-LATS1
LATS1
p-YAP
YAP
SHP2
p-CDC73
CDC73
GAPDH
Flag
MEIS2C/D
MEIS2A/B
C
HCC-LM3
MHCC97H
Sramble
siMEIS2C/D
siMEIS2C/D+antisi-FlagMEIS2C
siMEIS2C/D+antisi-FlagMEIS2D
OD 450nm Values
Days
D
HCC-LM3
MHCC97H
Ralative YAP mRNA levels
Ralative β-catenin levels
Scramble
siMEIS2C/D
siMEIS2C/D+antisi-FlagMEIS2C
siMEIS2C/D+antisi-FlagMEIS2D

Figure S5

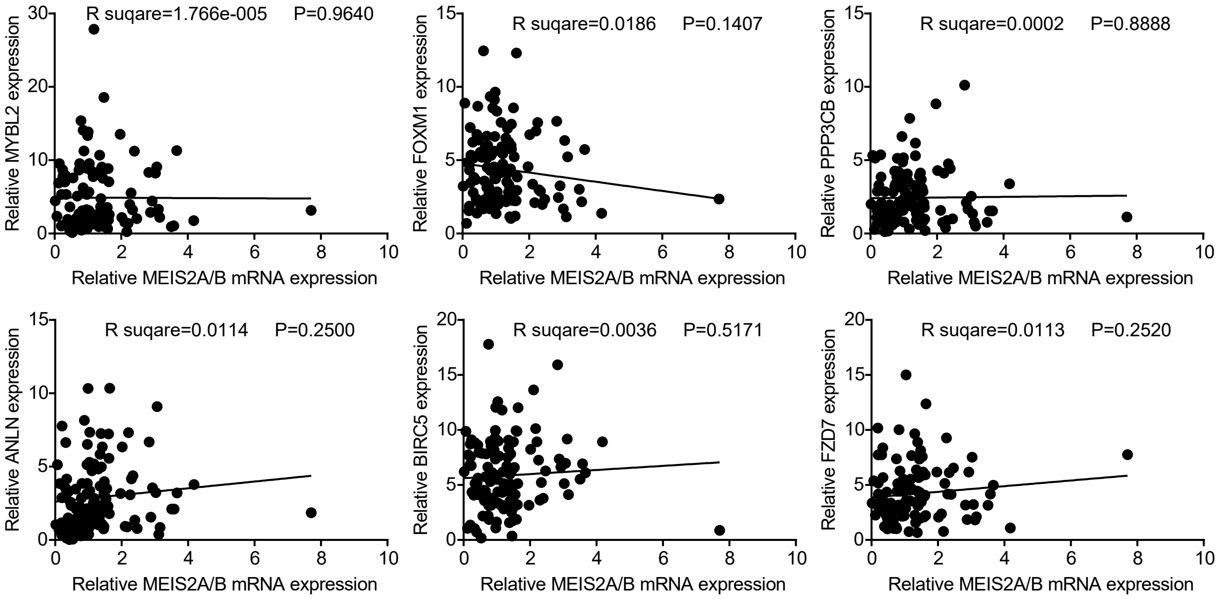
R suqare=1.766e-005 P=0.9640
Relative MYBL2 expression
Relative MEIS2A/B mRNA expression
R suqare=0.0186 P=0.1407
Relative FOXM1 expression
Relative MEIS2A/B mRNA expression
R suqare=0.0002 P=0.8888
Relative PPP3CB expression
Relative MEIS2A/B mRNA expression
R suqare=0.0114 P=0.2500
Relative ANLN expression
Relative MEIS2A/B mRNA expression
R suqare=0.0036 P=0.5171
Relative BIRC5 expression
Relative MEIS2A/B mRNA expression
R suqare=0.0113 P=0.2520
Relative FZD7 expression
Relative MEIS2A/B mRNA expression

Table S1

Characteristics	MEIS2C/D		
	Low(n=58)	High(n=60)	P-Value
Age,years,≤50:> 50	32:26	38:22	0.367
Gender, male:female	28:30	31:29	0.713
Serum AFP,μg/L,≤20:>20	39:19	43:17	0.602
HBsAg,Negative:Positive	33:25	40:20	0.275
Tumor capsule,No/incomplete:Complete	30:28	39:21	0.143
Tumor differentiation,Ⅰ-Ⅱ:Ⅲ-Ⅳ	21:37	7:53	0.002*
Vascular invasion,Yes:No	31:27	51:9	<0.001*
Liver cirrhosis,Yes:No	38:20	48:12	0.077
Tumor number,Solitary:Multiple	34:24	33:27	0.691
Tumour size, cm,≤5:>5	22:36	12:48	0.032*
TNM stage,Ⅰ:Ⅱ+Ⅲ	27:31	15:45	0.015*
BCLC stage,A:B+C	29:29	9:51	<0.001*

* represents P value with significant difference. P value was calculated by chi-square test or Fisher exact test.

Table S2

Model name	Start	End	Predicted site sequence
MEIS2	-2836	-2829	GTGACAGA
MEIS2	-2729	-2722	CTGACAGA
MEIS2	-2376	-2369	CTGACAGC
